# Divergent stage-specific regulation of neutrophil function by glucose transporter 1 in murine antibody-mediated glomerulonephritis

**DOI:** 10.1172/jci.insight.197169

**Published:** 2025-11-10

**Authors:** Hossein Rahimi, Wonseok Choi, Doureradjou Peroumal, Shuxia Wang, Partha S. Biswas

**Affiliations:** 1 Division of Rheumatology and Clinical Immunology, Department of Medicine, University of Pittsburgh, Pittsburgh, Pennsylvania, USA.; 2 Department of Microbiology and Immunology, Renaissance School of Medicine, Stony Brook University, SUNY, Stony Brook, New York, USA.

**Keywords:** Immunology, Inflammation, Nephrology, Innate immunity, Neutrophils

## Abstract

Prolonged and dysregulated neutrophilic inflammation causes tissue damage in chronic inflammatory diseases, including antibody-mediated glomerulonephritis (AGN). An increase in glycolysis, supported by enhanced glucose uptake, is a hallmark of hyperneutrophilic inflammation. Neutrophils upregulate glucose transporter 1–mediated (Glut1-mediated) glucose incorporation for renal antimicrobial activities. However, little is known about the role of neutrophil-specific Glut1 function in the pathogenesis of AGN. Using a well-vetted mouse model of AGN, we show that neutrophils upregulate Glut1 expression and function in the nephritic kidney. We demonstrate that Glut1 function in the hematopoietic cells during the early stage of the disease is necessary for kidney pathology. Most importantly, neutrophil-intrinsic Glut1 function is critical for AGN. While neutrophil-specific Glut1 ablation diminished the expression of tissue-damaging effector molecules in both the early and late stages, renal cytokines’ and chemokines’ production were compromised only in the late stage of the disease. Consequently, Glut1 inhibitor treatment ameliorated renal pathology in AGN mice. These data identify a Glut1-driven inflammatory circuit in neutrophils, which is amenable to therapeutic targeting in AGN.

## Abstract 2

This report describes how increased glucose metabolism drives the tissue-damaging function of neutrophils in chronic kidney diseases.

## Graphical abstract

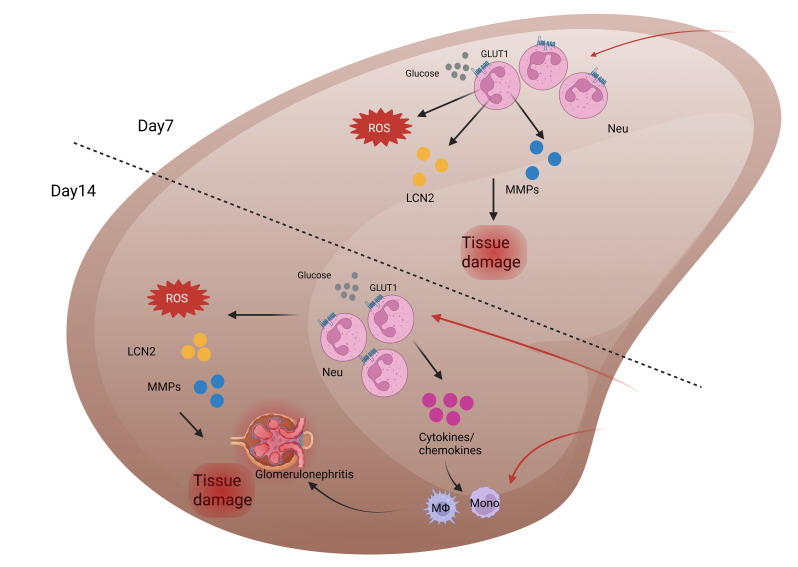

## Introduction

Immunometabolism plays a vital role in chronic inflammatory diseases, where prolonged and dysregulated immune system activation leads to metabolic reprogramming that exacerbates inflammation-mediated tissue damage (1–5). Chronic inflammation is driven in part by persistent metabolic reprogramming in immune cells. On the other hand, the inflammatory microenvironment controls several metabolic pathways that dictate the fate and function of immune effectors. Some chronic inflammatory diseases, including rheumatoid arthritis, systemic lupus erythematosus, and multiple sclerosis, have been shown to exacerbate in response to metabolic alterations and dysregulation of immune cells (1–5). Reprogramming immune response through metabolic changes has already shown promise in treating cancer (4). Four antiinflammatory drugs, including dimethyl fumarate, metformin, methotrexate, and rapamycin, regulate metabolism or mimic endogenous metabolites with antiinflammatory properties (4, 6). Thus, identifying metabolic changes as key contributors to unresolved inflammation may identify novel therapeutic targets in chronic inflammatory disorders.

A hallmark of many inflammatory cells is an increase in aerobic glycolysis rate fueled by increased uptake of extracellular glucose (1, 3). The inflammatory signals drive a metabolic switch in the immune cells, resulting in the upregulation of aerobic glycolysis, like the Warburg effect described in cancer cells. Indeed, glycolysis-dependent differentiation and function are apparent in activated M1 macrophages, monocytes, and NK, B, and T cells (4). Hence, limiting the rate of glycolysis provides a unique opportunity to shift the glycolytic flux, which is required to produce energy and excessive tissue-damaging effector molecules in immune cells. Consequently, inhibition of hexokinase by 2-DG and GAPDH by heptelidic acid has proven beneficial in controlling pathogenic inflammation (6). However, it is unknown whether blocking glucose transport by inhibiting glucose transporter 1 (Glut1) function will also hold similar promise as a therapeutic target to limit tissue damage in chronic inflammation.

Chronic kidney disease (CKD) is a significant public health concern because it causes a gradual loss of kidney function (7). The challenges in treating CKD revolve around preventing progression to irreversible end-stage kidney disease and managing complications, as there is no cure (7). Antibody-mediated glomerulonephritis (AGN) is a clinical manifestation of many forms of CKD, where the autoantibodies attack the glomerular basement membrane (GBM), leading to kidney damage and dysfunction (8, 9). The crescentic glomerulonephritis (GN), characterized by the formation of glomerular crescents and tubulointerstitial inflammation, is the most severe form of human AGN (10). The pathogenesis of AGN is poorly understood. Multiple studies using clinically relevant mouse models implicated the role of pro-inflammatory cytokine IL-17 in AGN (11–14). IL-17 produced by kidney-infiltrating innate and adaptive immune cells drives cytokines’ and chemokines’ production from the renal tubular epithelial cells (RTECs), which are critical for the migration of neutrophils in the nephritic kidney. Hyperneutrophilic glomerular and cortical inflammation, a hallmark of AGN, causes renal tissue damage by the exaggerated production of effector molecules, including reactive oxygen species (ROS), myeloperoxidase (MPO), matrix metalloproteinase (MMP), and neutrophil extracellular trap (NET) formation (15). Additionally, neutrophils produce cytokines and chemokines, required for the recruitment of other innate effector cells, capable of causing chronic inflammation and renal pathology. However, the role of Glut1-mediated glucose uptake in influencing neutrophil-mediated immunopathology in AGN is poorly understood.

Unlike most immune cell types, mitochondrial oxidative phosphorylation is dispensable for neutrophil function, thus making these cells metabolically unique (16–18). Hence, neutrophils rely on simpler metabolic pathways, such as glycolysis and the pentose phosphate pathway (PPP), to fulfill their energy requirements and support effector functions, including phagocytosis, degranulation, ROS production, and NET formation (19, 20). Individuals with rare genetic deficiency of glucose-6-phosphate (G6P) dehydrogenase or G6P transporter, key components of glycolysis and PPP, show increased susceptibility to infections (21, 22). Alternatively, inappropriate increase in glucose level or glycolysis fuels neutrophilic hyperinflammation, thus highlighting the importance of metabolic regulation of neutrophil function (23). Our published data showed that neutrophils respond to the metabolic demand by increased Glut1-mediated uptake of extracellular glucose (24). Here, we will define the role of neutrophil-intrinsic Glut1 function in the pathogenesis of AGN. We will also explore manipulating Glut1 function as a strategy to inhibit neutrophil-mediated tissue pathology in AGN.

In this report, we show that kidney-infiltrating neutrophils upregulate Glut1-mediated glucose uptake in the nephritic kidney. Glut1 expression in hematopoietic cells is indispensable for tissue pathology. Accordingly, Glut1 expression during the early phase of AGN development is critical for kidney dysfunction. Neutrophil-intrinsic Glut1 function, which is essential for AGN development, drives ROS, MMPs, and inflammatory mediators’ production in a stage-specific manner. Consequently, Glut1 inhibitor treatment ameliorates renal pathology in AGN mice. These findings demonstrate a potent, neutrophil-intrinsic metabolic reprogramming pathway that facilitates excessive glycolytic flux required for AGN development. This knowledge may allow the timely and selective targeting of metabolic pathways in neutrophils to prevent bystander tissue damage without compromising antimicrobial immunity during CKD.

## Results

### Neutrophils upregulate Glut1-mediated glucose uptake in the nephritic kidney.

**Figure 1:**
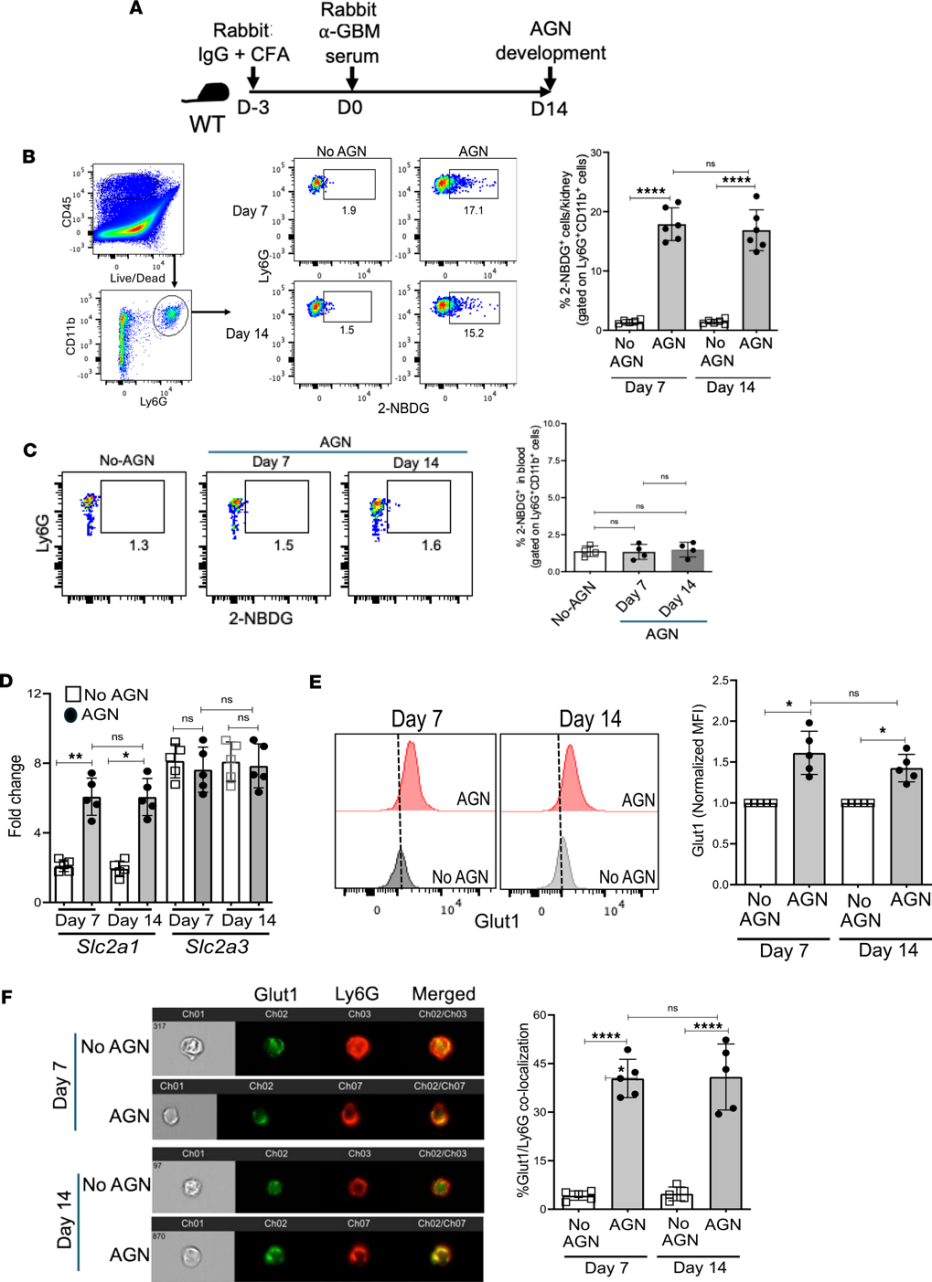
Kidney-infiltrating neutrophils upregulate Glut1-mediated glucose uptake in AGN. (**A**) Schematic representation of the mouse model of AGN. WT mice were subjected to AGN or left as no-AGN controls. At 7 and 14 days after anti-GBM serum injection, (**B**) kidney-infiltrating neutrophils (liveCD45^+^Ly6G^+^CD11b^+^) (*n* = 6) and (**C**) blood neutrophils (*n* = 4) were assessed for glucose uptake by 2-NBDG assay followed by flow cytometry. (**D**) Flow-sorted kidney-infiltrating neutrophils (*n* = 5) were evaluated for *Slc2a1* and *Slc2a3* transcript expression by reverse transcription quantitative PCR (RT-qPCR). (**E**) Glut1 protein expression in kidney-infiltrating neutrophils (*n* = 5) by intracellular staining followed by flow cytometry and (**F**) translocation of Glut1 protein from cytoplasm to the cell surface of kidney-infiltrating neutrophils (*n* = 5) by Cytek Biosciences ImageStream analysis. Data pooled from at least 2 independent studies (**B**–**F**). Each dot represents an individual mouse, and data are expressed as mean ± SD. Statistical analysis by 1-way ANOVA (**B**–**F**). *P < 0.05; **P < 0.01; ****P < 0.0001.

Hyperneutrophilic inflammation in the nephritic kidney is a hallmark of AGN (15). The tissue-damaging hyperneutrophilic response is a metabolically demanding process. The mechanisms by which neutrophils meet their high energy demand to execute exaggerated effector functions are poorly understood. To address this issue, we subjected wild-type (WT) mice to AGN by injecting them with rabbit IgG in complete Freund’s adjuvant (CFA), followed 3 days later by injection with rabbit anti-mouse GBM serum (24) (Figure 1A). We observed that glucose uptake was increased in kidney-infiltrating neutrophils but not blood neutrophils compared with non-AGN control mice at 7 (early time point) and 14 (late time point) days after anti-GBM serum injection, as measured by the incorporation of a fluorescent glucose analog, 2-NBDG (Figure 1, B and C). These data indicate that the renal inflammatory microenvironment plays a key role in regulating Glut1-mediated glucose uptake in neutrophils.

Most cells import glucose by facilitative diffusion across the cell membrane, a process mediated by the glucose transporter (Glut) family of membrane transport proteins (25). We showed that mouse neutrophils express only Glut1 (encoded by the *Slc2a1* gene) and Glut3 (encoded by the *Slc2a3* gene) at the baseline out of the 9 glucose transporters involved in glucose incorporation (24). The neutrophils upregulated Glut1 but not Glut3 transcripts and protein expression in the fungally infected kidney (24). Consistent with this observation, we observed that neutrophils exhibited increased *Slc2a1* transcript and Glut1 protein expression in the AGN kidney at 7 and 14 days after anti-GBM serum injection (Figure 1, D and E). In contrast, the Glut3 transcript was highly expressed at the baseline but remained unaltered in AGN. These data indicate that Glut1 is transcriptionally regulated in neutrophils in the AGN kidney.

Previous studies showed that Glut1 is stored in intracellular vesicles, and PKC-dependent phosphorylation of Glut1 is required for the surface localization and glucose transport (24, 26). We hypothesized that neutrophil activation in the inflamed kidney would lead to increased translocation of Glut1 from cytoplasmic vesicles to the cell membrane, which would aid in glucose uptake. The Amnis ImageStream analysis revealed an increase in Glut1 translocation to the cell membrane of kidney-infiltrating neutrophils at 7 and 14 days after anti-GBM injection (Figure 1F). These data indicate that kidney inflammation regulates transcription and cell membrane translocation of Glut1 in neutrophils during AGN.

### Glut1 plays a role in the pathogenesis of AGN.

**Figure 2:**
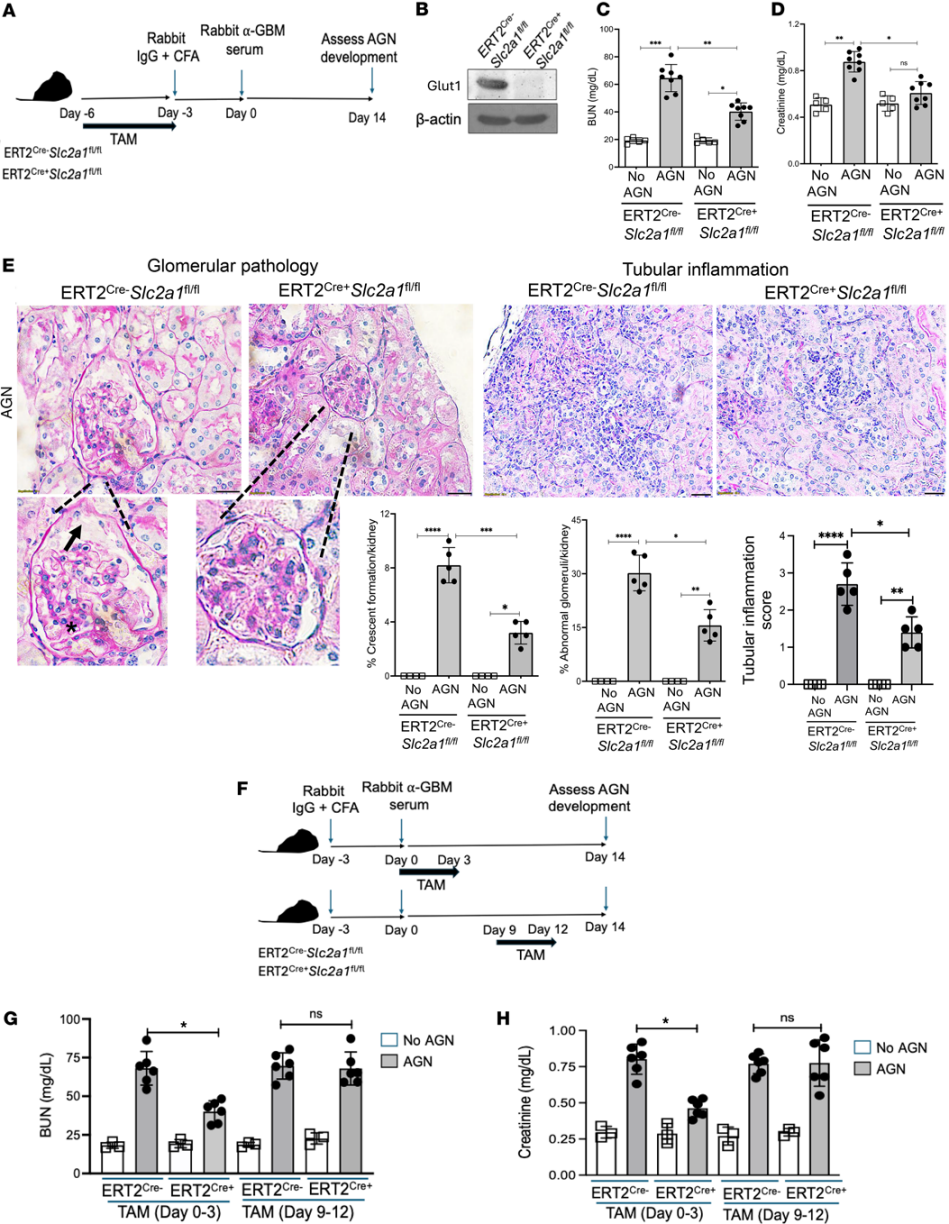
Glut1 plays a role in the pathogenesis of AGN. (**A**) Schematic diagram of the experimental design. Briefly, ERT2^Cre+^ *Slc2a1*^fl/fl^ and ERT2^Cre–^ *Slc2a1*^fl/fl^ mice were injected with tamoxifen (TAM) in corn oil for 4 consecutive days to delete the *Slc2a1* gene before subjecting them to AGN. (**B**) Kidneys (*n* = 3) were evaluated for Glut1 expression following TAM injection by immunoblot analysis. Mice were assessed for serum (**C**) BUN and (**D**) creatinine levels following AGN. (**E**) Representative photographs of periodic acid–Schiff–stained (PAS-stained) renal histopathology of kidney sections. Data representative 1 of 5 mice/group. Renal pathology was blindly evaluated and scored for percentages of abnormal glomeruli, crescent formation, and tubular inflammation. Magnification: glomerular pathology: 600×; tubular inflammation: 400×. A small part (as indicated by dotted lines) of the original image was shown as inset panels. Black arrow: Bowman’s capsule detachment; black star: mesangial cell proliferation and slight increase in cellularity. (**F**) Schematic diagram of the experimental plan. ERT2^Cre+^ *Slc2a1*^fl/fl^ and ERT2^Cre–^ *Slc2a1*^fl/fl^ mice were subjected to AGN. Mice received 4 consecutive injections of TAM during the early stage (days 0–3, relative to anti-GBM serum injection) or late stage (days 9–12, relative to anti-GBM serum injection) of the disease. Mice were evaluated for serum (**G**) BUN (*n* = 6) and (**H**) creatinine (*n* = 6) levels to measure kidney dysfunction. Data pooled from at least 2 independent studies (**C**–**E**, **G**, and **H**). Representative image from 1 of 3 independent experiments (**B**). Each dot represents an individual mouse, and data are expressed as mean ± SD. Statistical analysis by 1-way ANOVA (**B**–**D**, **F**, and **G**). *P < 0.05; **P < 0.01; ***P < 0.001; ****P < 0.0001.

Surprisingly little is known about the role of Glut1 in causing chronic kidney damage in AGN. Homozygous deletion of the *Slc2a1* gene results in embryonic lethality in mice (27). To overcome this technical limitation, we crossed *Slc2a1*-floxed mice to ERT2^Cre^ mice for spatiotemporal deletion of *Slc2a1* gene following tamoxifen administration. Accordingly, we efficiently deleted *Slc2a1* gene with tamoxifen injection daily for 4 days and subjected these mice to AGN (Figure 2, A and B). Mice with tamoxifen-induced conditional deletion of *Slc2a1* gene (ERT2^Cre+^ *Slc2a1*^fl/fl^) exhibited reduced kidney dysfunction following AGN, evidenced by diminished serum blood urea nitrogen (BUN) and creatinine levels compared with control mice (ERT2^Cre–^ *Slc2a1*^fl/fl^ receiving tamoxifen) (Figure 2, C and D). The ERT2^Cre+^ *Slc2a1*^fl/fl^ kidneys showed evidence of compromised glomerular and tubular pathology, as characterized by reduced crescent formation, mesangial hypercellularity, increased GBM thickness, and tubular inflammation (Figure 2E). These data indicate that Glut1 drives AGN pathogenesis, with the caveat that Glut1 is conditionally deleted before AGN.

We next sought to define at what stage of AGN development Glut1 expression is necessary and sufficient for kidney pathology. To explore this issue, *Slc2a1* gene was deleted by 4 consecutive tamoxifen injections at early (days 0–3) and late (days 9–12) stages, relative to anti-GBM serum injection, and we compared outcomes with those of control mice receiving tamoxifen (Figure 2F). Interestingly, the conditional deletion of *Slc2a1* gene at early but not late stages of the disease ameliorated kidney dysfunction in nephritic ERT2^Cre+^ *Slc2a1*^fl/fl^ mice (Figure 2, G and H). Overall, these results suggest that Glut1 expression and function during the early stages of AGN are essential for kidney inflammation and pathology.

### Mice with neutrophil-intrinsic deletion of Glut1 show ameliorated AGN.

**Figure 3:**
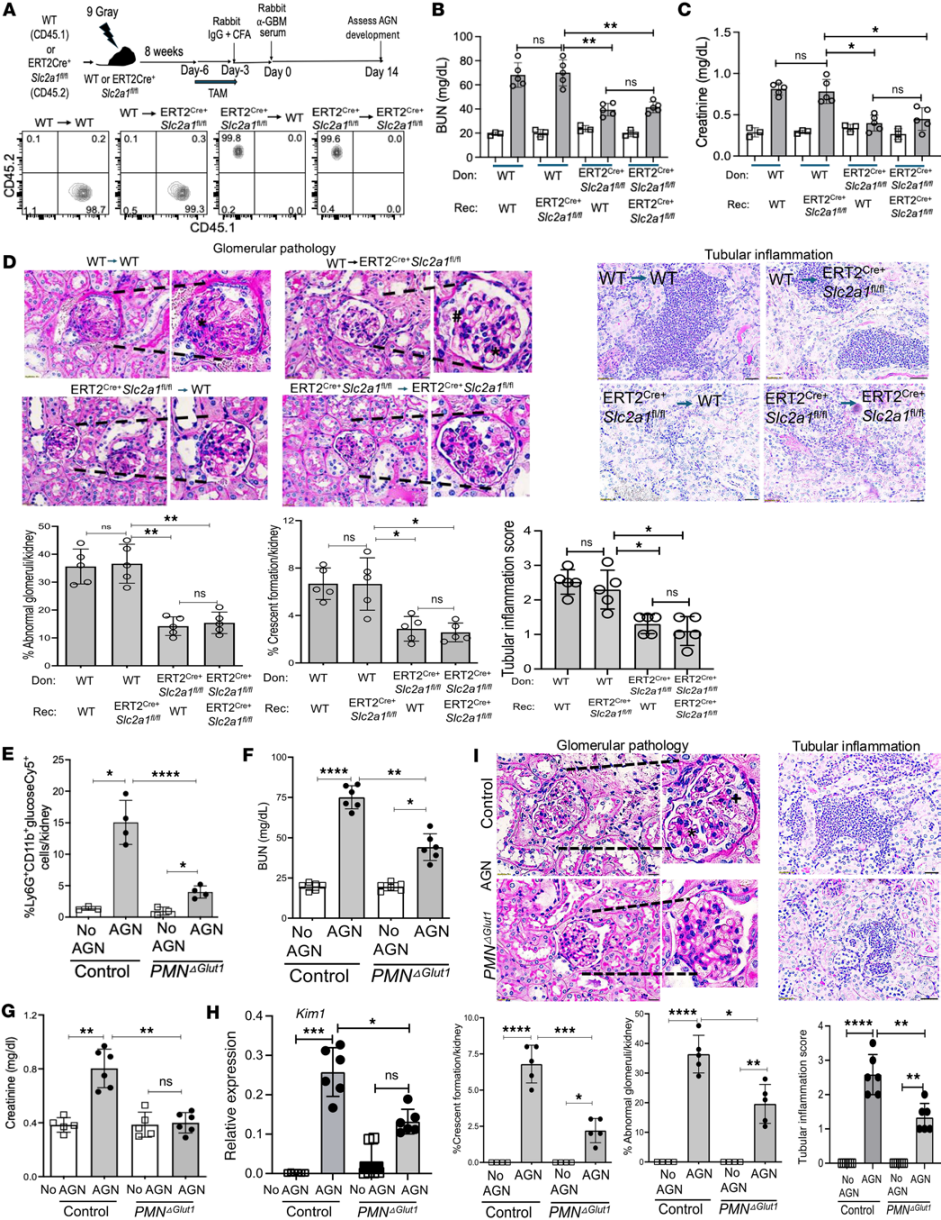
Neutrophil-specific Glut1 function reduces kidney damage in AGN. (**A**) Schematic diagram of the experiment. BM cells from ERT2^Cre+^ *Slc2a1*^fl/fl^ (CD45.2^+^) and WT (CD45.1^+^) mice were transferred into sublethally irradiated (9 Gy) ERT2^Cre+^ *Slc2a1*^fl/fl^ or WT recipients (*n* *=* 5). Eight weeks later, reconstituted mice were injected with 4 consecutive injections of TAM before being subjected to AGN. Representative flow cytometry contour plots showing reconstitution efficiency in the blood of recipient mice. At day 14 after anti-GBM serum injection, mice were assessed for kidney dysfunction by measuring serum (**B**) BUN and (**C**) creatinine levels. (**D**) Renal pathology (*n* = 5) was blindly evaluated and scored for percentages of abnormal glomeruli, crescent formation, and tubular inflammation following PAS staining of kidney sections. Data representative of 1 of 5 mice/group. Magnification: glomerular pathology: 600×; tubular inflammation: 400×. A small part (as indicated by dotted lines) of the original image was shown as inset panels. Black star: mesangial cell proliferation; black pound sign: glomerular atrophy. Control and *PMN^Glut1^* mice (*n* *=* 6) were subjected to AGN. At day 14 after anti-GBM serum injection, (**E**) in vivo glucose uptake, (**F**) serum BUN and (**G**) creatinine, and (**H**) renal *Kim1* mRNA levels were measured. (**I**) Renal pathology was evaluated and scored for percentages of abnormal glomeruli, crescent formation, and tubular inflammation following PAS staining. Data representative of 1 of 5 mice/group for I. Magnification: glomerular pathology: 600×; tubular inflammation: 400×. A small part (as indicated by dotted lines) of the original image was shown as inset panels. Black star: mesangial cell proliferation; black plus sign: partial segmental glomerulosclerosis. Data pooled from at least 2 independent studies (**B**–**I**). Each dot represents an individual mouse, and data are expressed as mean ± SD. Statistical analysis by 1-way ANOVA (**B**–**I**). *P < 0.05; **P < 0.01; ***P < 0.001; ****P < 0.0001.

Glut1 is expressed in both hematopoietic and nonhematopoietic cells. Both cell types contribute to the pathogenesis of CKD (25). To define the relative contribution of Glut1 in hematopoietic versus nonhematopoietic compartments, we created BM chimeric mice in which ERT2^Cre+^ *Slc2a1*^fl/fl^ (CD45.2) or WT (CD45.1) BM was adoptively transferred into irradiated reciprocal hosts. Successfully reconstituted recipients were then evaluated for susceptibility to AGN following 4 consecutive days of tamoxifen injection to delete Glut1 (Figure 3A). As shown in Figure 3B, ERT2^Cre+^ *Slc2a1*^fl/fl^ hosts receiving WT BM showed identical kidney dysfunction as WT mice receiving WT BM, indicating that Glut1 function in nonhematopoietic cells is dispensable for AGN pathology (Figure 3, B and C). In contrast, WT or ERT2^Cre+^ *Slc2a1*^fl/fl^ recipient mice receiving BM from ERT2^Cre+^ *Slc2a1*^fl/fl^ donors were resistant to AGN. Accordingly, WT or ERT2^Cre+^ *Slc2a1*^fl/fl^ hosts reconstituted with ERT2^Cre+^ *Slc2a1*^fl/fl^ donor BM showed diminished kidney pathology and inflammation (Figure 3D). Thus, Glut1 in the hematopoietic cells is required for the development of AGN.

The neutrophils are the primary hematopoietic kidney-infiltrating cells that play an important role in AGN (28). We showed that neutrophils upregulate Glut1-mediated glucose uptake during neutrophilic hyperinflammation in AGN (Figure 1). Based on these observations, we sought to define the role of Glut1 in neutrophils following AGN. To that end, we crossed *Slc2a1^fl/fl^* mice to *MRP8^Cre^* mice (termed *PMN^Glut1^*), which selectively and efficiently depleted Glut1 in neutrophils, as previously reported (24). The *MRP8^Cre+^* *Slc2a*^fl–/fl–^ mice (henceforth referred to as control) were used as controls. The kidney-infiltrating neutrophils of *PMN^Glut1^* mice demonstrated a defect in glucose uptake following AGN (Figure 3E). Following AGN, *PMN^Glut1^* mice showed diminished levels of serum BUN and creatinine in the serum, indicating ameliorated kidney disease (Figure 3, F and G). Accordingly, the kidneys from *PMN^Glut1^* mice revealed reduced kidney injury marker *Kim1* mRNA expression, glomerular crescent formation, mesangial hypercellularity, increased GBM thickness, and tubular inflammation in comparison with the control kidney (Figure 3, H and I). These results highlight a potentially previously unrecognized neutrophil-specific role of Glut1 in AGN pathology.

### Neutrophil-specific Glut1 expression affects local inflammatory response in the kidney.

**Figure 4:**
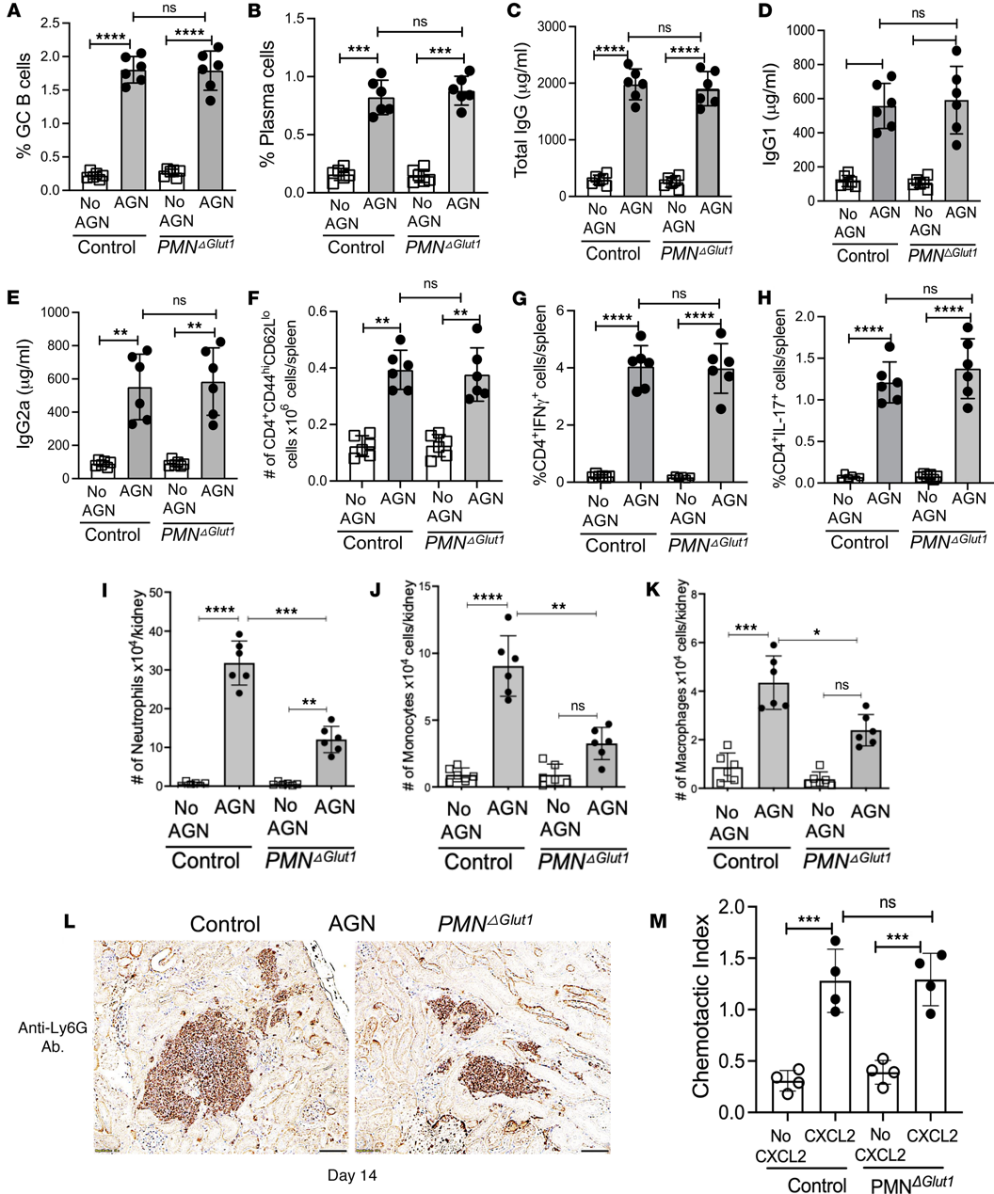
*PMN^Glut1^* mice show compromised kidney inflammatory response in AGN. Control and *PMN^Glut1^* mice (*n* = 6) were subjected to AGN. (**A**) Flow cytometric analysis of splenic cells from control and *PMN^Glut1^* mice (*n* *=* 6) was performed at day 12 after rabbit IgG + CFA immunization to determine the frequency of (**A**) GC B cells (liveB220^+^GL-7^+^CD95^+^) and (**B**) plasma cells (liveB220^lo^CD138^hi^). At day 12, serum titers of mouse anti-rabbit (**C**) total IgG, (**D**) IgG1 isotype, and (**E**) IgG2a isotype were measured by ELISA. Flow cytometric analysis of splenic cells from control and *PMN^Glut1^* mice (*n* *=* 6) was performed at day 12 to determine the frequency of (**F**) effector CD4^+^ T cells (liveCD4^+^CD62L^lo^CD44^hi^). The splenocytes were stimulated with PMA/ionomycin for 4 hours and intracellularly stained for (**G**) IFN-γ and (**H**) IL-17 followed by flow cytometry analyses (gated on CD4^+^ cells). At day 14 after anti-GBM serum injection, control and *PMN^Glut1^* kidneys (*n* = 6) were assessed for kidney-infiltrating (**I**) neutrophils (liveCD45^+^Ly6G^+^CD11b^+^), (**J**) monocytes (liveCD45^+^Ly6G^-^CD11c^+^CD11b^hi^F4/80^lo^), and (**K**) macrophages (liveCD45^+^Ly6G^–^CD11c^+^CD11b^lo^F4/80^hi^) by flow cytometry. (**L**) At day 14 after anti-GBM serum injection, kidneys were subjected to immunohistochemistry using anti-Ly6G antibody. Image representative of 1 of 5 mice/group. Magnification: 400×. (**M**) *PMN^Glut1^* and control BM neutrophils were subjected to Transwell plate migration assay in the presence or absence of CXCL2 (500 ng/mL) for 30 minutes. The number of neutrophils was counted in the lower and upper chambers and expressed as the Chemotactic Index (number of cells in the lower chamber/number of cells in the upper chamber). Data pooled from at least 2 (**A**–**K**) and 4 independent studies (**L**). Each dot represents an individual mouse (**A**–**K**) and individual experiments (**M**), and data are expressed as mean ± SD. Statistical analysis by 1-way ANOVA (**A**–**K** and **M**). *P < 0.05; **P < 0.01; ***P < 0.001; ****P < 0.0001.

The lack of clinical disease in *PMN^Glut1^* mice may reflect either an inability of Glut1-deficient neutrophils to support systemic immune response or a block in their ability to drive chronic inflammation in the inflamed kidney or both. While neutrophils are primarily known for their role in innate immunity, they can also present antigens to B and T cells in secondary lymphoid organs and regulate adaptive immune responses (29, 30). Hence, to assess whether the lack of Glut1 expression in neutrophils affects humoral immunity, we measured B cell response in the *PMN^Glut1^* spleen following immunization with rabbit IgG + CFA. As shown in Figure 4, A and B, percentages of germinal center B cells and plasma cells were comparable between the control and *PMN^Glut1^* spleens. We also analyzed the total IgG and isotype pattern of mouse IgG antibody response directed against rabbit IgG in the serum of control and *PMN^Glut1^* mice. There was no difference in the total mouse IgG antibody titers to rabbit IgG between the groups (Figure 4C). Moreover, no bias for Th1 (IgG2a) or Th2 (IgG1) antibody production was observed in the absence of Glut1 in the neutrophils (Figure 4, D and E).

Th cells play a dominant role in AGN (31, 32). The spleens of control and *PMN^Glut1^* mice demonstrated a similar number of activated CD4^+^ T cells following AGN (Figure 4F). When evaluated for effector cytokine production by CD4^+^ T cells, we observed a similar number of IFN-γ^+^ and IL-17^+^CD4^+^ T cells in the spleens of control and *PMN^Glut1^* mice (Figure 4, G and H). These data suggest that systemic B and T cell responses were not affected when Glut1 was deleted specifically in neutrophils in AGN.

Next, to evaluate the contribution of neutrophil-specific Glut1 expression in local inflammatory reaction in the kidney, we determined the frequency of kidney-infiltrating innate effectors at day 14 after anti-GBM serum injection. The number of kidney-infiltrating total neutrophils, and inflammatory monocytes and macrophages, was reduced in the absence of Glut1 in neutrophils (Figure 4, I–K). Accordingly, immunohistochemistry analysis revealed diminished neutrophil accumulation in the *PMN^Glut1^* mice (Figure 4L). Moreover, BM neutrophils from the *PMN^Glut1^* and control mice exhibited comparable in vitro CXCL2-driven migration, as revealed by Transwell plate migration assay (Figure 4M). These results indicate a role for neutrophil-specific Glut1 expression as a positive regulator of local inflammatory response in the nephritic kidney.

### Glut1 differentially regulates the pathogenic effector function of neutrophils at different stages of AGN.

**Figure 5:**
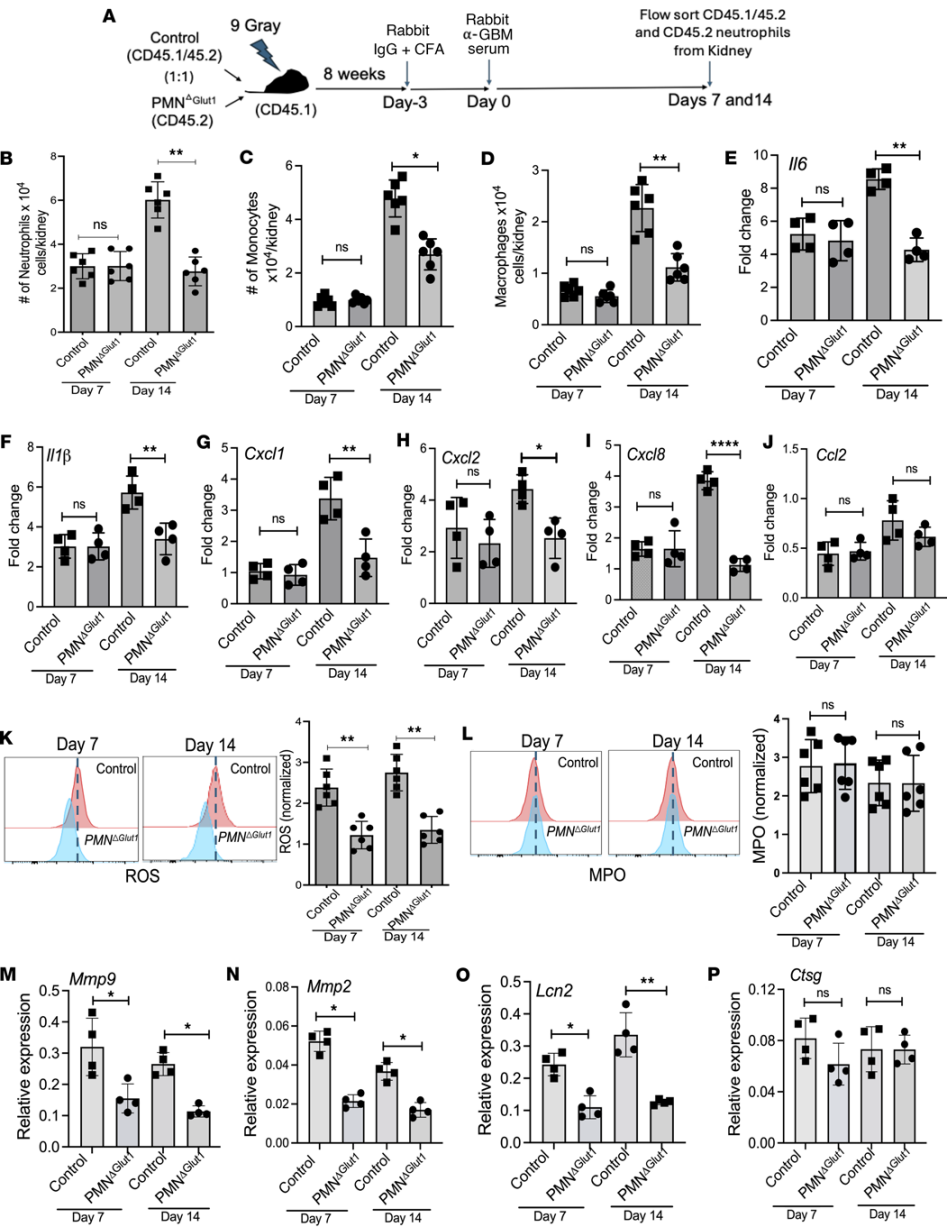
Diminished expression of inflammatory and tissue-damaging factors by neutrophils depleted of Glut1 function. (**A**) Irradiated (9 Gy) C57BL/6 (CD45.1) mice were reconstituted with control (CD45.1/1.2) and *PMN^Glut1^* (CD45.2) mixed BM (1:1). Eight weeks later, successfully reconstituted mice were subjected to AGN. Kidneys (*n* = 6) were evaluated for the total number of (**B**) neutrophils (liveCD45^+^Ly6G^+^CD11b^+^), (**C**) monocytes (liveCD45^+^Ly6G^-^CD11c^+^CD11b^hi^F4/80^lo^), and (**D**) macrophages (liveCD45^+^Ly6G^–^CD11c^+^CD11b^lo^F4/80^hi^) by flow cytometry. At 7 and 14 days after anti-GBM serum injection, FACS-sorted kidney-infiltrating neutrophils (*n* = 4) were evaluated for (**E**–**J**) *Il6*, *Il1β*, *Cxcl1*, *Cxcl2*, *Cxcl8*, and *Ccl2* transcript expression by RT-qPCR (*n* = 4), (**K**) ROS (*n* = 6) and (**L**) preformed MPO expression (*n* = 6) by flow cytometry (*n* = 6), and (**M**–**P**) *Mmp9*, *Mmp2*, *Lcn2*, and *Ctsg* transcript expression by flow-sorted neutrophils (*n* = 4) by RT-qPCR (*n* = 4). Data pooled from at least 2 independent studies (**B**–**P**). Each dot represents an individual mouse, and data are expressed as mean ± SD. Statistical analysis by 1-way ANOVA (**B**–**P**). *P < 0.05; **P < 0.01; ****P < 0.0001.

We next sought to determine how Glut1 contributes to the pathogenic function of neutrophils in the AGN kidney. The amount of inflammation and tissue damage negatively affects the level of tissue glucose. Therefore, it is important to exclude the confounding effect of the difference in renal inflammation and tissue glucose level on glucose availability to kidney-infiltrating neutrophils. To achieve this objective, irradiated WT (CD45.2) mice were co-reconstituted with control (CD45.1/45.2) and *PMN^Glut1^* BM (CD45.2) (mixed BM chimera — 1:1) and subjected to AGN (Figure 5A). This allowed us to perform a head-to-head comparison between WT and *PMN^Glut1^* neutrophil function within the same tissue microenvironment. Although comparable infiltration of neutrophils, monocytes, and macrophages was noted in the nephritic kidney at early time point (day 7), the influx of neutrophils lacking Glut1 was markedly compromised at the late time point (day 14) (Figure 5B). We also observed a reduction in the number of monocytes and macrophages at the late time point, indicating that Glut1 function in neutrophils directly or indirectly regulates tissue migration of other innate inflammatory cells (Figure 5, C and D).

The neutrophils produce cytokines and chemokines to attract immune cells with potent antimicrobial and tissue-damaging properties. To determine whether the diminished number of infiltrating innate effectors correlates with reduced cytokine and chemokine production by Glut1-depleted neutrophils, we measured the transcript expression of various cytokines and chemokines genes in flow-sorted neutrophils (>95% purity, based on congenic markers) from the nephritic kidney at early and late time points. In line with the cell infiltration data (Figure 5, B–D), there was no difference in the mRNA expression of *Il6*, *Il1β*, *Cxcl1*, *Cxcl2*, *Cxcl8*, and *Ccl2* between WT and *PMN^Glut1^* neutrophils at the early time point (day 7) (Figure 5, E–J). However, at a late time point (day 14), *PMN^Glut1^* neutrophils showed diminished expression of *Il6*, *Il1β*, *Cxcl1*, *Cxcl2*, and *Cxcl8* transcripts compared with WT neutrophils. There was no difference in the *Ccl2* mRNA expression between the groups. Collectively, these data suggest a role for neutrophil-specific Glut1 function in driving the expression of multiple cytokine and chemokine genes at the late stage of the disease, which is required for the infiltration of neutrophils and other innate effectors in the AGN kidney.

In addition to aiding in the tissue migration of immune cells, neutrophils can cause tissue damage by releasing toxic substances like ROS, proteolytic enzymes, and antimicrobial peptides, as well as by forming NETs (28, 33). In contrast with cytokine and chemokine gene expression, Glut1-ablated neutrophils demonstrated a defect in ROS production in both the early and late stages of AGN development (Figure 5K). Intriguingly, there was no impairment in the preformed MPO expression by neutrophils between the groups (Figure 5L). When evaluated for proteases and antimicrobial peptides, flow-sorted *PMN^Glut1^* neutrophils exhibited a profound defect in *Mmp9*, *Mmp2* (matrix-degrading proteases), and *Lcn2* (antimicrobial peptide) transcript expression at both the early and late time points (Figure 5, M–O). There was comparable expression of *Ctsg* (gene encoding antimicrobial peptide cathepsin G) between the groups (Figure 5P). These data indicate that increased tissue damage by Glut1-dependent MMPs and ROS in the early stage of AGN initiates an inflammatory circuit, which fuels the exaggerated production of cytokines from neutrophils, a process required for inflammatory cell influx and tissue damage in the late stage of the disease.

### Glut1 blockade shows preclinical therapeutic efficacy in AGN.

**Figure 6:**
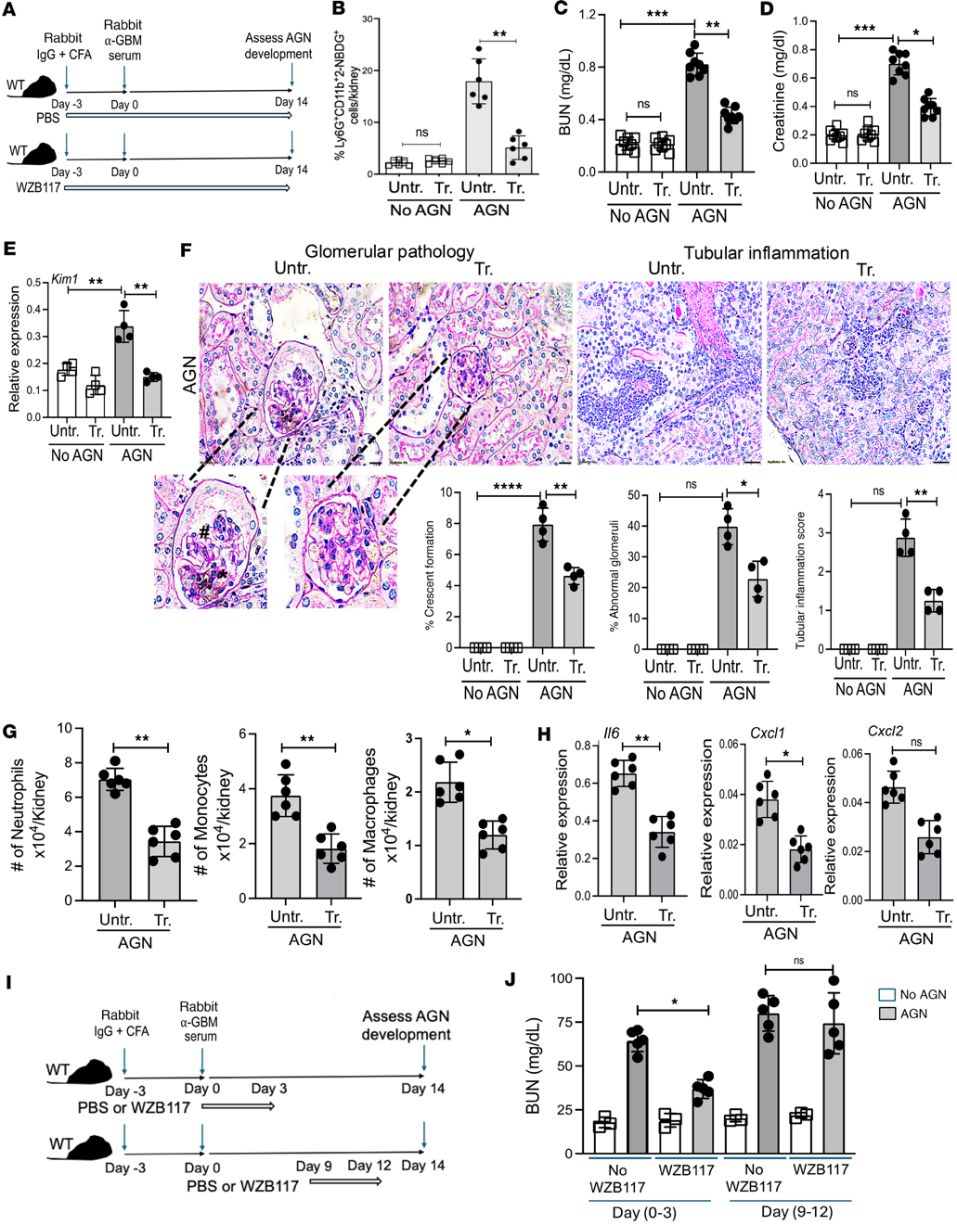
Glut1 inhibitor treatment ameliorates renal pathology in AGN. (**A**) Schematic diagram of the experiment. Groups of WT mice were subjected to AGN and either treated with WZB117 (Glut1 inhibitor: 10 mg/kg/d i.p. injection) daily starting day –3 (relative to anti-GBM serum injection) and then daily for the next 14 days or left untreated. (**B**) At day 7, glucose uptake by neutrophils in the kidney was measured. At day 14, mice were evaluated for serum (**C**) BUN (*n* = 8), (**D**) creatinine level (*n* = 8), and (**E**) *Kim1* transcript expression in the kidney (*n* = 4). (**F**) Kidney histopathology was blindly evaluated and scored following PAS staining. Data representative of 1 of 4 mice/group. Magnification: glomerular pathology: 600×; tubular inflammation: 400×. A small part (as indicated by dotted lines) of the original image was shown as inset panels. Black star: mesangial cell proliferation; black pound sign: glomerular atrophy. (**G**) Flow cytometric analysis was performed at day 14 (*n* = 6) to determine the number of neutrophils (liveCD45^+^Ly6G^+^CD11b^+^Ly6C^−^), inflammatory monocytes (liveCD45^+^Ly6G^−^Ly6C^+^CD11b^hi^F4/80^lo^), and macrophages (liveCD45^+^Ly6G^−^Ly6C^+^CD11b^lo^F4/80^hi^). (**H**) The transcript expression of *Il6*, *Cxcl1*, and *Cxcl2* in the nephritic kidneys (*n* = 6) was measured by RT-qPCR at day 14 after anti-GBM serum injection. (**I**) Schematic diagram of the experiment. WT mice were subjected to AGN and either treated with WZB117 daily days 0 to 3 (relative to anti-GBM serum injection) or days 9–12 or left untreated (*n* = 5). (**J**) At day 14, mice were evaluated for serum BUN. Data pooled from at least 2 independent studies (**B**–**H** and **J**). Each dot represents an individual mouse, and data are expressed as mean ± SD. Statistical analysis by 1-way ANOVA (**B**–**F** and **J**) and 2-tailed Student’s *t* test (**G** and **H**). *P < 0.05; **P < 0.01; ***P < 0.001; ****P < 0.0001.

Therapeutic targeting of Glut1-mediated glucose uptake using small-molecule inhibitors has shown promise in treating cancer (34, 35). Hence, we assessed the preclinical therapeutic efficacy of the specific Glut1 inhibitor WZB117 in treating AGN in mice. To that end, WT mice were either treated with WZB117 (i.p. injection) starting day –3 and then daily for the next 17 days after rabbit IgG + CFA immunization or left untreated (Figure 6A). As expected, WZB117 treatment compromised glucose uptake by kidney-infiltrating neutrophils following AGN (Figure 6B). Mice treated with WZB117 showed diminished levels of serum BUN and creatinine compared with untreated mice (Figure 6, C and D). Consequently, WZB117 treatment reduced *Kim1* mRNA expression, renal inflammation, and pathology in the AGN kidney (Figure 6, E and F). When assessed for effector function, neutrophils from WZB117-treated mice showed diminished inflammatory cell, cytokine, and chemokine transcripts compared with untreated kidneys (Figure 6, G and H).

Based on our results in Figure 2, E–G, we also treated WT mice with Glut1-specific inhibitor (WZB117) during the early or late stages of the disease and assessed kidney dysfunction following AGN (Figure 6I). Supporting our previous data (Figure 2, E–G), Glut1 inhibitor treatment during the early but not the late stage of the disease reduced kidney dysfunction in AGN mice (Figure 6J). These results highlight the beneficial effect of blocking Glut1 function during the early stages of the disease in mice subjected to AGN.

## Discussion

Inflammation is a physiological and highly regulated response to injury and infection to regain homeostasis (36). This is primarily achieved by balancing the production of pro- and antiinflammatory mediators. However, unresolved or chronic inflammation tips the balance toward a pro-inflammatory phenotype and thus ensuing immunopathology and end-organ damage. Unregulated neutrophil function has been implicated in many inflammatory diseases, including CKD (15). Glut1 is a major glucose transporter in neutrophils, and Glut1-mediated glucose transport across the cell membrane is important for their activity (24). On the other hand, an inappropriate increase in the glucose level, or glycolysis, fuels neutrophilic hyperinflammation (23). By taking advantage of a heterologous mouse model of AGN, we showed that neutrophil-intrinsic Glut1 function drives pathogenic neutrophilic response by increasing the pro-inflammatory profile and redox status in a stage-specific manner. We acknowledge that using an autologous model of AGN without preimmunization would have been more suitable to study hyperneutrophilic response in CKD. However, the custom-prepared mouse anti-GBM serum used in the present study, as well as in previous reports from our laboratory, induces a weak AGN without pre-immunization. Additionally, a lack of metabolic caging facilities made measuring urinary albumin in mice challenging because of difficulties in obtaining uncontaminated and sufficient urine samples. Hence, we relied on serum BUN and creatinine measurements to assess kidney function in AGN mice. Nevertheless, knowledge gained from this study may allow the timely and selective targeting of metabolic pathways to prevent bystander tissue damage without compromising the antimicrobial function of neutrophils.

Glucose transporters play an important role in kidney function, including the release of glucose from gluconeogenesis and the reabsorption of glucose (37, 38). The expression of multiple Glut proteins has been identified in the kidney. Glut1 is one of the most abundantly expressed isoforms, primarily in the proximal tubule (38). Most of the studies focused on understanding the role of Glut1 in the RTECs in diabetic kidney disease, where Glut1 expression and function are altered, potentially contributing to the disease’s development. Despite their well-characterized role in kidney-resident cells in diabetic nephropathy, Glut1 function in the kidney-infiltrating immune cells is entirely undefined. Most importantly, no studies to date have focused on how Glut1 regulates the pathogenic function of neutrophils in CKD. We show that the contribution of Glut1 function in the nonhematopoietic system in AGN pathogenesis is, in fact, negligible. Rather, Glut1 function in hematopoietic cells is critical to drive the production of pathogenic factors, including cytokines, chemokines, and tissue-damaging molecules in the inflamed kidney. However, we do not rule out the indirect impact of Glut1 deletion in neutrophils on the function of kidney-resident nonhematopoietic cells in AGN. Glut1-regulated inflammatory and tissue-damaging effector molecules from neutrophils may directly impact glomerular and tubular cells’ survival and function, which warrants careful future investigation. The neutrophils also express Glut3 under homeostatic conditions. Although Glut3 is implicated in neutrophil development, there is no evidence suggesting a pathogenic role of Glut3 in AGN development. The findings in this study, thus, set the stage for pursuing an analysis of these related but poorly understood Gluts in neutrophils in CKD, which may have ramifications for other hyperneutrophilic inflammatory diseases.

The contribution of kidney-infiltrating monocytes, macrophages, dendritic cells, and kidney-resident macrophages is often overlooked in the context of AGN pathogenesis. Unlike neutrophils, which express Glut1 and Glut3 only, these innate cells are equipped with multiple Gluts to facilitate glucose transport during inflammation. Glut1 overexpression in a macrophage cell line confers a hyperinflammatory phenotype via increased production of ROS and pro-inflammatory cytokine production, underscoring the importance of Glut1 function in these innate cell types in driving CKD (39, 40). However, kidney dysfunction as measured by serum BUN and creatinine values was comparable between the neutrophil-specific and tamoxifen-inducible global Glut1-knockout mice, thus arguing against the contribution of Glut1 function in other kidney-infiltrating innate effectors in the renal pathology. Nevertheless, using cell type–specific conditional knockout mice to define the relative contribution of Glut1 in different innate cell populations in AGN warrants careful investigation.

Neutrophils are confirmed as an effector cell in regulating B cell–mediated antibody response, which is critical for AGN pathogenesis (41). Indeed, neutrophils can internalize and process antigens, then present them on MHC class II molecules, potentially influencing B cell responses. In macrophages, the Glut1 function affects antigen presentation by regulating autophagy, a process linked to antigen processing and presentation (39, 40). In contrast, our data show that the absence of Glut1 in neutrophils has no negative effect on germinal center formation and antibody response following immunization with rabbit IgG and CFA. These data point to a critical and likely direct role for neutrophil-specific Glut1 function in driving local inflammatory response in the nephritic kidney. Interestingly, the immunohistochemistry for neutrophils reveals extensive infiltration in the tubulointerstitial compartment rather than within the glomeruli, suggesting tubular injury or inflammation precede glomerular injury, a hallmark of glomerulonephritis.

Our study showed that Glut1-mediated glucose uptake regulates the gene expression of inflammatory mediators and tissue-damaging effector molecules in a stage-specific manner. The underlying signaling mechanisms of how Glut1 controls gene expression in neutrophils are poorly understood. One possible explanation is that Glut1-mediated glucose uptake fuels the energy-demanding cellular processes required for gene transcription and protein translation in neutrophils. However, a previous study showed that Glut1 can also activate a pro-inflammatory profile through NF-κB in macrophages (40). Consequently, silencing Glut1 expression in macrophages inhibited LPS-induced IκB degradation, thus blocking NF-κB activation. Additionally, it is possible Glut1 function in neutrophils upregulates autophagy, affecting various pro-inflammatory functions like phagocytosis, degranulation, and NETosis in the nephritic kidney. Hence, future studies will need to address how Glut1 function in neutrophils drives pro-inflammatory phenotype in AGN.

Reprogramming immune response by targeting Glut1-dependent glucose uptake has already shown promise in treating cancer (42). Selective inhibition of Glut1 function reportedly exhibited antitumor effects by suppressing cancer cell proliferation. However, efforts to target Glut1 function in chronic inflammatory diseases, including CKD, have not been studied in detail. Glut1-haplodeficient mice showed ameliorated retinal pathophysiology in diabetic mice (43). Our data suggest that targeting the Glut1 pathway may be a safe, inexpensive, and rapidly implementable treatment option for AGN, since highly selective Glut1 inhibitors, such as BAY-876, are currently in use for preclinical studies in cancer (42). Moreover, inhibiting sodium-glucose cotransporter 2 (SGLT2), which plays a critical role in glucose reabsorption, has shown clinical efficacy in treating CKD, especially in patients with diabetes (44). Thus, dual targeting of Glut1 and SGLT2 may be a potential therapeutic approach in the treatment of AGN and other CKD. These studies may justify future preclinical studies of Glut1 inhibitors to treat or prevent CKD, with broader implications for other hyperneutrophilic inflammatory conditions.

## Methods

### Sex as a biological variable.

All the experiments used age-matched controls of both sexes. Data from different sexes were pooled.

### Mice.

C57BL/6 (WT) mice were purchased from Taconic Biosciences Inc. ERT2^Cre^ and MRP8^Cre^ mice were purchased from Jackson Laboratory. *Slc2a1*^fl/fl^ mice are under a Material Transfer Agreement from the University of Colorado, USA. To generate mice with conditional deletion of Glut1 in neutrophils, we crossed the *Slc2a1*^fl/fl^ mice with ERT2^Cre^ or MRP8^Cre^ IRES/GFP mice. B6.SJL-Ptprc (B6 CD45.1) were from Jackson Laboratory. MRP8^Cre+^ *Slc2a1^fl–/fl–^* (CD45.2) mice were crossed to B6 CD45.1 to generate MRP8^Cre+^ *Slc2a1^fl–/fl–^* (CD45.1/45.2) animals. All mice were housed in specific pathogen–free conditions under the supervision of the Division of Laboratory Animal Resources, University of Pittsburgh, and Stony Brook University. Mice from different facilities were cohoused for at least 3 weeks to normalize microbiota.

### Mouse model of AGN.

Mice were immunized i.p. with 0.2 mg of rabbit IgG (Jackson ImmunoResearch catalog 011-000-003) in CFA (MilliporeSigma). Controls received CFA only. Three days later, mice were injected i.v. with heat-inactivated rabbit anti–mouse GBM serum (custom-made, Lampire Biological Lab Inc.) at 5 mg/20 g body weight. Mice were sacrificed at days 7 or 14 after anti-GBM injection. Blood was obtained by retro-orbital bleeding.

### BM chimeras.

To create BM chimeras, mice were sublethally irradiated (9 Gy). Twenty-four hours later, 5 × 10^6^ to 10 × 10^6^ donor BM cells (either CD45.1 or CD45.2) were injected i.v. For mixed BM chimeras, 5 × 10^6^ to 10 × 10^6^ donor BM cells of CD45.1/45.2 and CD45.2 origin were adoptively transferred in 1:1 ratio. After 8 weeks, peripheral blood of recipients was tested for successful reconstitution with donor BM cells by flow cytometry for CD45.1 and CD45.2 (LSRFortessa cytometer, BD Biosciences).

### Kidney function test.

Serum BUN was measured using Blood Urea Nitrogen Enzymatic kit (Bioo Scientific Corp.) and creatinine with the QuantiChrom Creatinine Assay kit (BioAssay Systems).

### Glucose uptake measurement.

For glucose uptake measurement in kidney-infiltrating neutrophils, mice were subjected to AGN. At days 7 and 14 after anti-GBM serum injection, mice were injected i.v. with 200 μL of 2-(N-(7-Nitrobenz-2-oxa-1,3-diazol-4-yl)Amino)-2-Deoxyglucose (2-NBDG) or GlucoseCy5 (provided by Greg M. Delgoffe, University of Pittsburgh) in PBS. Thirty minutes later, kidney-infiltrating neutrophils were analyzed for glucose uptake by flow cytometry.

### ImageStream analysis.

For Glut1 localization in kidney-infiltrating neutrophils, mice were subjected to AGN. At days 7 and 14 after anti-GBM serum injection, the kidney single-cell suspension was stained with anti-Ly6G antibody (eBioscience, clone IA8), followed by intracellular staining with anti-Glut1 antibody (Abcam, clone EPR3915). The colocalization of Ly6G and Glut1 fluorescence was detected by Amnis ImageStream analysis (Cytek Biosciences) and expressed as a percentage of surface-expressed (Ly6G colocalized) Glut1 out of total Glut1 protein.

### RNA extraction and RT-qPCR.

Total RNA was extracted from tissues or neutrophils using RNeasy kits (QIAGEN). Complementary DNA was synthesized by SuperScript III First Strand Kits (Thermo Fisher Scientific). Quantitative real-time PCR was performed with the PerfeCTa SYBR Green FastMix (Quanta BioSciences) and analyzed on an Applied Biosystems ABI 7300 real-time instrument. Primers were obtained from QuantiTect Primer Assays (QIAGEN). The expression of each gene was normalized to that of *Gapdh*.

### Histopathology.

For histopathology, kidneys were fixed with 10% buffered formaldehyde and embedded in paraffin. Slices of 4 μm thickness were stained with PAS and observed on an EVOS microscope (Thermo Fisher Scientific). The slides were scored blindly by an expert with more than 10 years of experience with renal histopathology, as described previously (24). Briefly, the severity of GN was assessed by a mild to moderate increase in mesangial cellularity, thickening of the GBM, endocapillary hypercellularity, and crescent formation. The tubulointerstitial inflammation was measured by assessing tubular atrophy and tubulointerstitial inflammation.

### Immunohistochemistry.

Immunohistochemistry staining was done on formalin-fixed, paraffin-embedded sections. Sections were rehydrated, and antigen retrieval was performed with heated citrate. Primary antibody against Ly6G (Abcam) was used. Secondary antibodies used were biotinylated anti-rabbit IgG antibodies (provided in VECTASTAIN ABC-HRP Kit; catalog PK-4001, Vector Laboratories). ABC-HRP Kit and DAB reagent (Vector Laboratories) were used to develop colors. The sections were counterstained with hematoxylin and visualized using an Olympus CKX41 microscope.

### Flow cytometry.

Single-cell suspensions from tissues were prepared as previously described (24). Briefly, spleens were subjected to mechanical dissociation followed by red blood cell lysis. Kidneys were perfused with PBS containing EDTA (MilliporeSigma) before harvesting. Kidneys were digested at 37°C in 1 mg/mL collagenase IV (Worthington) in complete RPMI for 30 minutes, filtered through 70 µm strainers (Thermo Fisher Scientific), and washed twice in PBS. For flow cytometry, the following antibodies were used: CD45 (30-F11, eBioscience), CD45R/B220 (RA3-6B2, BioLegend), Ly6G (IA8, eBioscience), CD11b (M1/70, BioLegend), F4/80 (BM8, BioLegend), Ly6C (HK1.4, eBioscience), CD45.1 (A20, BioLegend), CD45.2 (104, BioLegend), GL-7 (GL7, BD Biosciences), CD95 (15A7, Invitrogen), CD138 (281-2, BD Biosciences), CD4 (GK1.5, BioLegend), CD44 (IM7, Invitrogen), CD62L (MEL-14, BD Biosciences), Glut1 (EPR3915, Abcam), IFN-γ (XMG1.2, BioLegend), and IL-17 (TC11-18H19, BD Pharmingen). The cells were fixed and permeabilized before intracellular staining. Dead cells were excluded using the Live/Dead Ghost Dye Violet 510 dye. Samples were acquired on LSR Fortessa cytometer (BD Biosciences) and analyzed by FlowJo software (Tree Star Inc.).

### ROS measurement.

Kidney cell suspensions were prepared and resuspended in RPMI complete medium. CellROX Deep Red reagent was added to the cell suspensions and incubated at 37°C for 30 minutes. The ROS production was measured by flow cytometry analysis.

### Transwell plate migration assay.

BM neutrophils were subjected to Transwell migration assay (Nunc Thermo Fisher Scientific polycarbonate inserts with 3 μm pore size) in the presence of recombinant murine CXCL2 (500 ng/mL) (PeproTech). After 30 minutes, the number of cells in the lower and upper chambers was quantified by flow cytometry and expressed as Chemotactic Index (Chemotactic Index: number of cells in the lower chamber/number of cells in the upper chamber).

### Injection of tamoxifen.

Mice were i.p. injected with tamoxifen (3 mg) in corn oil once daily for 4 days.

### Treatment with a Glut1 inhibitor.

Mice were i.p. injected with Glut1 inhibitor WZB117 (Sigma) (10 mg/kg/d i.p.) or vehicle control (45).

### Statistics.

All data are shown as mean ± SD. Statistical analyses were performed using unpaired 2-tailed Student’s *t* test and 1-way ANOVA, depending on the experiment, using GraphPad Prism. A *P* value less than 0.05 was considered statistically significant. All experiments were performed at least twice in independent replicates.

### Study approval.

The research described here complies with all relevant ethical regulations. All the experiments were conducted following NIH *Guide for the Care and Use of Laboratory Animals* (National Academies Press, 2011) under protocols approved by the University of Pittsburgh IACUC (protocol no. 20087922) and SUNY, Stony Brook IACUC (protocol no. 2024-00044).

### Data availability.

The data underlying Figures 1–6 are available in the published article and Supporting Data Values file.

## Author contributions

HR and PSB designed the experiments, and HR, WC, DP, SW, and PSB performed the experiments. HR, WC, and PSB analyzed and interpreted the data, and PSB wrote the manuscript.

## Funding support

This work is the result of NIH funding, in whole or in part, and is subject to the NIH Public Access Policy. Through acceptance of this federal funding, the NIH has been given a right to make the work publicly available in PubMed Central.

- NIH grants AI159058, AI142354, and AI181831 to PSB.

## Supplementary Material

Unedited blot and gel images

Supporting data values

## Version 1 11/10/2025

Electronic publication
