# Supplementary figures and images for: Divergent stage-specific regulation of neutrophil function by glucose transporter 1 in murine antibody-mediated glomerulonephritis
Source: JCI Insight. 2025 Nov 10;10(21):e197169. doi: 10.1172/jci.insight.197169 (PMC12643513; doi:10.1172/jci.insight.197169)

## Unedited immunoblot images of Fig 2B

Glut1

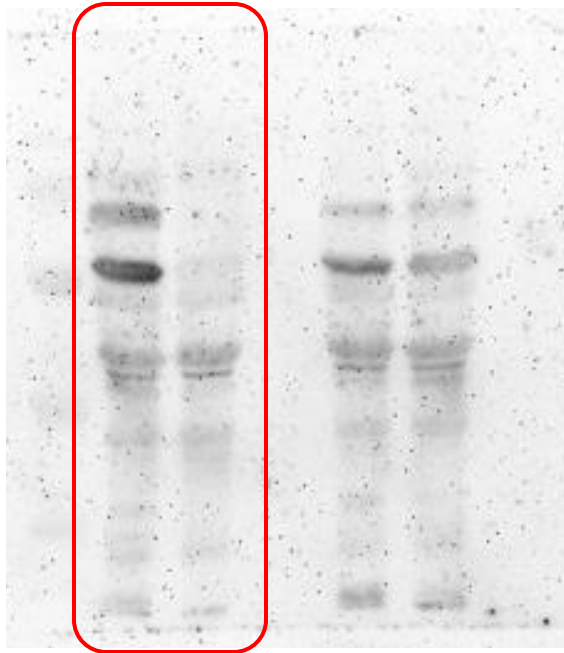

$\beta$ -actin

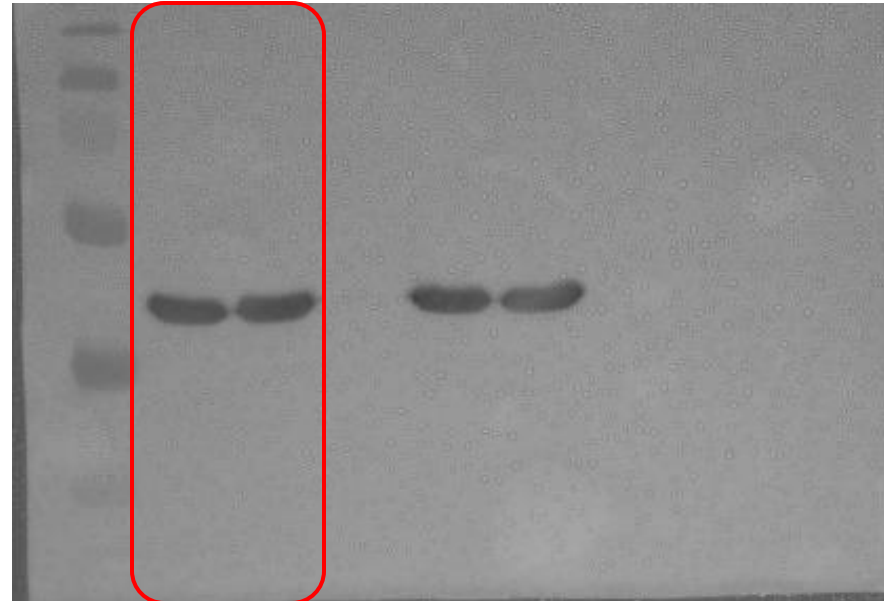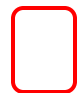

Lanes used for Fig 2B

Supplement: Unedited blot and gel images [file jciinsight-10-197169-s194.pdf]
